# Neurological outcome after emergency radiotherapy in MSCC of patients with non-small cell lung cancer - a prospective trial

**DOI:** 10.1186/1748-717X-8-297

**Published:** 2013-12-28

**Authors:** Harald Rief, Rita C Heinhold, Lina C Petersen, Stefan Rieken, Thomas Bruckner, Arash Moghaddam-Alvandi, Jürgen Debus, Florian Sterzing

**Affiliations:** 1 Department of Radiation Oncology, University Hospital of Heidelberg, Im Neuenheimer Feld 400, 69120 Heidelberg, Germany; 2 Department of Medical Biometry, University Hospital of Heidelberg, Im Neuenheimer Feld 305, 69120 Heidelberg, Germany; 3 Department of Orthopaedics, Trauma & Paraplegiologie, University Hospital of Heidelberg, Schlierbacher Landstraße 200a, 69118 Heidelberg, Germany

**Keywords:** Emergency radiotherapy, MSCC, Spine, Neurological outcome

## Abstract

**Background:**

The aim of this trial was to investigate neurological outcome after emergency RT in MSCC of NSCLC patients with acute neurological deficit.

**Methods:**

This pilot trial was prospective, non-randomized, and monocentre, ten patients were treated from July 2012 until June 2013. After onset of neurological symptoms RT was started within 12 hours. The neurological outcome was assessed at baseline, and six weeks after RT using the ASIA Impairment Scale (AIS).

**Results:**

The results showed an improved neurological outcome in one patient (10%), one patient (10%) had a decreased, and five patients (50%) a constant outcome after six weeks. Three patients (30%) died within the first six weeks following RT, additional 4 patients (40%) died within 4 month due to tumor progression.

**Conclusion:**

In this group of NSCLC patients we were able to show that emergency RT in MSCC with acute neurological deficit had no considerable benefit in neurological outcome. Therefore, short-course regime or best supportive care due to poor survival should be considered for these patients with additional distant metastases. Patients with favorable prognosis may be candidates for long-course RT.

**Trial Registration:**

Clinical trial identifier NCT 02000518.

## Introduction

Malignant spinal cord compression (MSCC) is secondary to bone metastases to the vertebral column associated with mechanical compression of the myelon [1]. MSCC occurs in around 5% of terminal cancer patients within the last 2 years of life [2], lung cancer accounts for 15-20% of cases [3]. MSCC is a frequent medical emergency, and the most common treatment offered is radiotherapy. Treatment goals include maintenance of neurological function, control of local tumor growth, spine stabilization, and pain control [4]. Acute neurological deficit in MSCC is an emergency condition in radiation oncology. Without treatment, the spinal cord can be irreversibly damaged resulting in deteriorating or permanent sensorimotor deficits. Radiotherapy (RT) was shown to be fast, time sparing, and a very effective treatment option for MSCC [5]. Despite some reports about the high efficacy of radiation treatment for oncological emergencies, a standard of care is not well defined, especially the time interval of immediate RT after deficit, and neurological outcome with respect to poor survival. The objective of our trial was to investigate neurological outcome after emergency RT in MSCC of non-small cell lung cancer (NSCLC) patients with acute neurological deficit.

## Methods

### Subjects and recruitment

**Figure 1:**
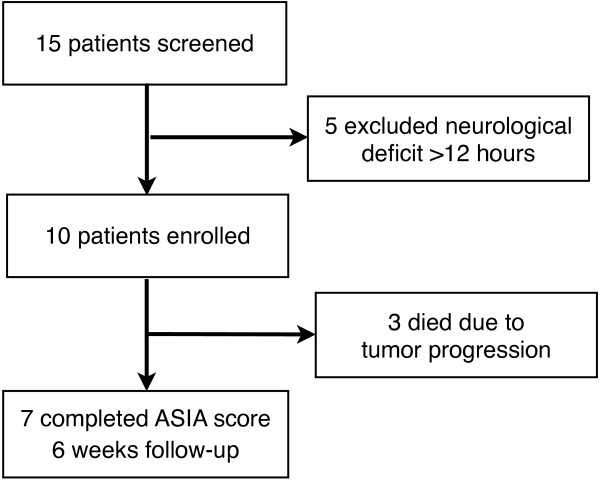
Flow of participants through the trial.

From July 2012 until June 2013, 15 consecutive patients with histologically confirmed non-small cell lung cancer and bone metastases of the thoracic or lumbar segments of the vertebral column were screened for this trial in the Radiooncology Department of the Heidelberg University Clinic. The patients were subjected to a staging of their vertebral column within the context of the computer tomography scans (CT) and magnetic resonance imaging (MRI) designed to plan the RT schedule prior to enrolment into the trial. Inclusion criteria were an age of 18 to 80 years, acute neurological symptom caused by MSCC, no RT in this spinal area before, and written consent to participate. Out of 15 patients considered eligible 5 patients were excluded due to neurological deficit confirmed longer than 12 hours. The remaining 10 patients fulfilled the inclusion and were enrolled into the trial (Figure 1). The study was approved by the Heidelberg Ethics Committee (Nr. S-514/2011).

### Design and procedures

This is a prospective, controlled, explorative trial to investigate the neurological outcome after emergency RT in MSCC of NSCLC patients with acute neurological deficit. RT started within 12 hours after onset of neurological symptoms. The target parameters were measured at the start of radiotherapy (t_0_), and six weeks after RT (t_1_). The target parameters comprise the documentation and completion of the ASIA Impairment Scale (AIS), and the recording of patient-specific data. The data of the patient records were collected by the authors. Patient characteristics are shown in Table 1.

**Table 1:** Patient characteristics at baseline

		**n**	**%**
Age (years)			
	Mean (SD)	58 (11)	
Gender			
	Male	9	90
	Female	1	10
KPS (median, range)		55 (50–70)	
Histology	Adenocarcinoma	9	90
	Squamous cell carcinoma	1	10
Localization metastases			
	Thoracic	7	70
	Lumbar	3	30
Number metastases			
	Mean (range)	2 (1–5)	
	Solitary	3	30
	Multiple	7	70
Type of metastases			
	Mixed	1	10
	Osteolytic	9	90
Distant metastases at baseline			
	Visceral	5	50
	Brain	3	30
	Lung	4	40
Ambulatory status prior to RT			
	Not ambulatory	9	90
	Ambulatory before RT	1	10
Immunotherapy		2	20
Chemotherapy		7	70
Pathological fracture at baseline		0	0
Radiotherapy schedule (Gy)			
Single dose (median, range)		3 (2.5-3)	
Cumulative dose (median, range)		30 (30–35)	

SD Standard deviation, RT Radiotherapy.

The primary endpoint was neurological outcome, assessed using the ASIA Impairment Scale (AIS), which is specially designed to standard neurological classification for patients with spinal cord injury [6]. In sensory level, the light touch and pin prick (right and left) was tested in each dermatomes (0 = absent, 1 = impaired, 2 = normal). The total of light touch and pin prick was 112 pts. left and right each. In motor level, the upper limb and lower limb total was 50 pts. left and right each. The muscle grading was defined 0 = total paralysis, 1 = palpable or visible contraction, 2 = active movement, full range of motion, gravity eliminated, 3 = active movement, full range of motion, against gravity, 4 = active movement, full range of motion, against gravity and provides some resistance, 5 = active movement, full range of motion, against gravity and provides normal resistance. The survival was defined as begin RT (begin neurological symptom) to death.

Radiotherapy was performed in the Radiooncology Department of the Heidelberg University Clinic. After virtual simulation was performed to plan the radiation schedule, radiotherapy was carried out over a dorsal photon field of the 6MV energy range. PTV covered the specific vertebral body affected as well as the ones immediately above and below. 8 patients (80%) were treated with 10 × 3 Gy, 2 patients (20%) with 14 x 2.5 Gy. The median individual dose in all patients was 3 Gy (range 2.5-3 Gy), the median total dose 30 Gy (range 30–35 Gy). The individual and total doses were decided separately for each individual patient.

All variables were analyzed descriptively by tabulation of the measures of the empirical distributions. According to the scale level of the variables, means, standard deviations, medians as well as minimum and maximum or absolute and relative frequencies, respectively, will be reported.

## Results

The median follow-up was 2.1 months. During the trial there were no adverse events and no dropouts. The median Karnofsky performance score (KPS) was 55, only one (10%) patient had a pre-radiotherapy ambulatory status.

According the ASIA score, one patient (10%) improved the neurological outcome, one patient (10%) had a decreased, and five patients (50%) a constant outcome after six weeks. The motor and sensory score were considerably similar after six weeks, and summarized in Tables 2 and 3.

**Table 2:** Five categories of the ASIA impairment scale

**A**	Complete. No sensory or motor function
	is preserved in the sacral segments S4-S5.
**B**	Sensory Incomplete. Sensory but not
	motor function is preserved below the
	neurological level and includes the sacral
	segments S4-S5 (light touch, pin prick at S4-S5:
	or deep anal pressure (DAP)), and no motor
	function is preserved more than three levels below
	the motor level on either side of the body.
**C**	Motor Incomplete. Motor function is
	preserved below the neurological level, and
	more than half of key muscle functions below the
	single neurological level of injury (NLI) have a
	muscle grade less than 3 (Grades 0–2).
**D**	Motor Incomplete. Motor function is
	preserved below the neurological level, and at
	least half (half or more) of key muscle functions
	below the NLI have a muscle grade > 3.
**E**	Normal. If sensation and motor function
	are graded as normal in all segments, and the
	patient had prior deficits, then the AIS grade is E.

**Table 3:** Results of ASIA score at baseline and after 6 weeks

**Motor**	**Baseline**					**6 weeks**				
	**Mean**	**SD**	**Min**	**Max**		**Mean**	**SD**	**Min**	**Max**	
Upper limb	48	4.3	38	50		47	7.9	29	50	
Lower limb	25	18.3	10	50		30	18.4	14	50	
Voluntary anal contraction	n = 4 (40%)					n = 4 (40%)				
Sensory	Baseline					6 weeks				
	Mean	SD	Min	Max		Mean	SD	Min	Max	
Light touch	82	21.8	50	108		76	23.2	53	108	
Pin prick	74	24.9	42	107		73	29.1	51	106	
AIS	A	B	C	D	E	A	B	C	D	E
	3	0	4	3	0	2	0	2	3	0

AIS ASIA Impairment Scale.

**Figure 2:**
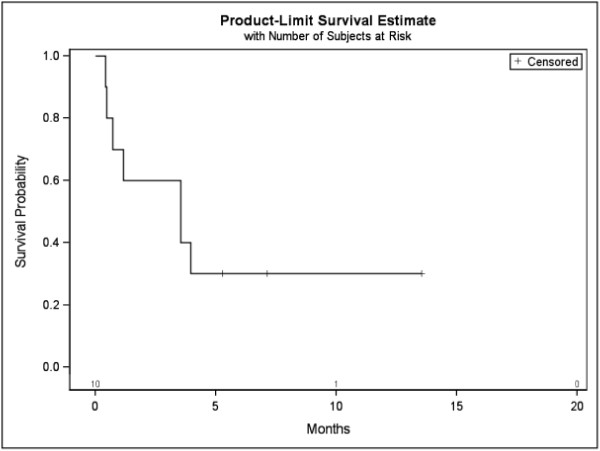
Kaplan-Meier curve for survival (begin neurological symptom to death).

Three patients (30%) died within the first six weeks following RT, additional 4 patients (40%) died within 4 month due to tumor progression. The median survival time was 2.57 month (SD = 0.53) from begin RT to death (Figure 2). We found three groups with different survival after RT. The three cases with rapid deaths were compared to brain and/or visceral metastases. The group of patients (n = 4) who died within 4 months was compared to progression of primary site, one (10%) decreased in neurological outcome. In all cases, the number or spinal localization of metastases did not correlate with survival. In 3 patients, survival was longer than 4 month, all of them had no other distant metastases, and one improved the neurological outcome. The chemotherapy or immunotherapy before RT showed no influence in survival.

## Discussion

Acute neurological deficit in MSCC is an emergency condition in radiation oncology. A rapidly developed neurological symptom in MSCC is a rare condition, most of symptoms develop slowly. RT and surgery are the most frequently applied treatment modalities [1]. RT is the most commonly used option due to poor prognosis in lung cancer [7]. Time factor of begin RT (emergency RT within 12 hours) after neurological symptom cause of MSCC is not well defined. Our results showed no considerable benefit in neurological outcome after emergency RT in MSCC of NSCLC patients with acute neurological deficit. Only one patient (10%) improved to outcome after 6 weeks. Christian et al. showed in 1033 patients, that emergency RT produces only 50% improvement rate [5], however this was a result of different primary tumour sites. Another work of Rades et al. in 84 patients presented an improvement of motor function occurred in 24% after radiotherapy [8]. The emergency RT in MSCC of NSCLC patients with acute neurological deficit is very rare, therefore our sample size was low. Nevertheless this prospective trial presented first results of neurological outcome according the ASIA score after emergency RT. Previous trials showed no detriment to functional outcome and response rate in different radiotherapy schedules [9-11]. The schedules in our trial were decided separately for each individual patient. The survival of patients with MSCC depends on the primary tumour, lung cancer is generally dismal [1]. The life expectancy of most MSCC patients is quite short, with reported median survival of only few months [12]. In our trial, 7 patients (70%) died within 4 months after RT. These patients may be candidates for short-course RT, single fraction RT, or even best supportive care alone. In the retrospective study of Rades et al. 1852 patients with different tumours the factors predicting for improved survival were female gender, a favourable histology, the absence of visceral or other extensive bone metastases, a good performance status, being ambulatory and a slower development of motor deficits before radiotherapy [1]. All these predicting factors were in our short-survival group absent. We were not able to show predicting factors due the small sample size. In this unfavourable group of patients, the optimal dose would be just a single 8 Gy dose, as it has been shown to provide equivalent functional recovery, local control and survival compared with longer schedules [13]. In three cases (30%) of our participants, survival was longer than 4 months. These patients had no other distant metastases, and one improved the neurological outcome, and may be candidates for long-course RT such as 10 × 3 Gy or 14 × 2.5 Gy for better local control. Validated scoring systems are needed to identify long-term survivors for decision of fractionation [14,15]. Estimating the prognosis is critical to achieving a balance between effective therapy and the burden of treatment. Treatment can be individualized by reviewing simple prognosis scales. For patients with a poor prognosis, a single fraction of 8 Gy or 5 × 4 Gy is just as effective as multiple fractions and much more convenient [16].

Most institutions give corticosteroids in addition to radiation treatment, which is strongly recommended in the literature [17]. In the present study, all patients were treated with corticosteroids 24 mg per day.

Limitations of our trial were the single arm design and low number of participants. The strength of our work comprised the first performance of emergency RT within 12 hours after neurological symptom and prospective assessment of neurological outcome according to a validated score.

## Conclusion

In this group of NSCLC patients we were able to show that emergency RT in MSCC with acute neurological deficit had no considerable benefit in neurological outcome. Short-course regimens or best supportive care due to poor survival should be considered for these patients with additional distant metastases. Patients with favorable prognosis may be candidates for long-course RT.

## Competing interests

The authors declare that they have no competing interests.

## Authors’ contributions

HR and AM developed and planned this trial. TB is responsible for statistical considerations/basis of the analysis. HR, SR, FS, and JD performed the examinations and RT supervisions. HR made the data collection. All authors read and approved the final manuscript.
